# Mental health, work and healthcare: shared responsibilities for well-being

**DOI:** 10.1192/bji.2026.10134

**Published:** 2026-11

**Authors:** Hussien Elkholy

**Affiliations:** 1 Department of Clinical Neuroscience, Brighton and Sussex Medical School, University of Brighton and University of Sussex, Brighton, UK; 2 Sussex Partnership NHS Foundation Trust, Worthing, UK; 3 Neurology and Psychiatry Department, Faculty of Medicine, Ain Shams University, Cairo, Egypt.

The health and well-being of employees are integral to the functioning and productivity of any organisation. This includes mental health, which is a leading cause of long-term sickness absence.^1,2^ Because health and well-being at work are interrelated and share overlapping determinants, they need to be approached holistically rather than as separate concerns. The articles in this issue suggest a connected picture. Despite growing evidence on mental health problems in the workplace and on potential interventions, there remains a notable gap in implementation and organisational accountability.^3^ Across young adulthood, professional life, clinical assessment and service delivery, well-being emerges not simply as a personal resource to be cultivated but as an outcome shaped by the environments in which people study, work, seek help and provide care.

Depression and anxiety are usually discussed not only as factors affecting performance and attendance, but also as possible outcomes of unfavourable working conditions. Witte and colleagues explored longitudinal associations between perceived occupational challenges and depressive symptoms among middle-aged German workers, situating mental health within the changing realities of employment, responsibility and healthy work environments.^4^ Alpona et al^5^ extended this discussion to physicians, examining how work characteristics and mental health may intersect with gendered experiences and expectations. Read together, these articles challenge any simple separation between mental health and work. They point instead to the need for workplaces that recognise psychological strain early, reduce avoidable harms and make well-being part of everyday organisational practice.

On a different, but relevant, note, healthcare workforces provide a particularly urgent example of this wider challenge. The discussion of the UK’s addiction medical workforce crisis moves the issue from individual well-being to service sustainability.^6^ When services are under strain, the consequences extend beyond their impact on individual clinicians: they affect access, continuity, quality and the capacity of systems to respond to complex need. In this sense, workforce well-being is not a secondary concern within mental health services: it is part of the infrastructure of care itself.

Mental health is sustained not only through personal skills and relationships, but also through fair working conditions, culturally responsive assessment, accessible services and interventions that address people’s lives and needs. If workplace well-being is to be meaningful, it must be understood not as a benefit or add-on, but as a central part of public mental health, clinical care and organisational responsibility.

This issue also includes articles addressing resilience in young adulthood, cognitive assessment in diverse populations and lifestyle interventions for people with severe mental illness. Together, they broaden the conversation from workplace well-being to the wider conditions that support mental health across settings. I hope you will enjoy reading it.
